# Association of Nutrient-, Food-, and Lifestyle-Based Oxidative Balance Scores with Liver Enzymes in Older Adults Across Cardiovascular-Risk Groups

**DOI:** 10.3390/antiox15091077

**Published:** 2026-08-28

**Authors:** Hawa Sidibé, Mojgan Morvaridzadeh, Tamàs Fülöp, Hicham Berrougui, Slimane Belbraouet, Michel Nguyen, Abdelouahed Khalil

**Affiliations:** 1 Geriatrics Unit, Department of Medicine, Faculty of Medicine and Health Sciences, University of Sherbrooke, Sherbrooke, QC J1H 5N4, Canada; hawa.sidibe2@usherbrooke.ca (H.S.); mojgan.morvaridzadeh@usherbrooke.ca (M.M.); tamas.fulop@usherbrooke.ca (T.F.);; 2 Ecole de Nutrition, Université de Moncton, Moncton, NB E1A 3E9, Canada; slimane.belbraouet@umoncton.ca; 3 Cardiology Unit, Department of Medicine, Faculty of Medicine and Health Sciences, University of Sherbrooke, Sherbrooke, QC J1H 5N4, Canada; michel.nguyen@usherbrooke.ca

**Keywords:** cardiovascular risk, oxidative balance score, HDL cholesterol, liver transaminases, older adults

## Abstract

Diet and lifestyle are modifiable determinants of oxidative balance through their influence on exposure to antioxidant and pro-oxidant factors. The oxidative balance score (OBS) is a composite index that reflects the balance between these exposures, with higher scores indicating a predominance of antioxidant factors. In this exploratory pilot study, we examined three OBSs (nutrient-, food-, and lifestyle-based), as well as their combined versions (nutrient–lifestyle and food–lifestyle), across clinical subgroups and evaluated their associations with biomarkers of oxidative stress, inflammation, lipid metabolism, and liver-related biomarkers. A total of 44 older adults were enrolled and stratified into three subgroups (16 healthy, 14 hypercholesterolemia, and 14 post–myocardial infarction). Each participant completed a questionnaire, a 3-day food record, and underwent blood sampling. OBSs were calculated based on 15 nutrients, nine food groups, and two lifestyle components. Correlations and multiple linear regression analyses were performed to examine associations between OBSs and the following biomarkers: plasma total antioxidant capacity (TEAC and FRAP), C-reactive protein (CRP), HDL cholesterol, alanine aminotransferase (ALT), and aspartate aminotransferase (AST). The nutrient–lifestyle OBS (OBSN-L) was significantly associated with lower ALT and AST (adjusted ALT β = −1.06; *p* = 0.048; AST β = −0.54; *p* = 0.034). A similar association was observed for the nutrient OBS (OBSN) (adjusted AST β = −0.64; *p* = 0.022). No significant associations were observed for TEAC, FRAP, or CRP. Neither the OBSF nor the OBSF-L was associated with any circulating biomarkers. Higher nutrient-based OBSs, with or without lifestyle integration, were independently associated with lower liver transaminase levels in older adults with varying levels of cardiovascular risk. OBSs may help to capture dietary and lifestyle patterns associated with liver-related biomarkers. These exploratory findings warrant confirmation in larger, prospective studies.

## 1. Introduction

Cardiovascular diseases (CVDs) remain the leading cause of morbidity and mortality worldwide [1]. Oxidative stress plays a central role in the pathophysiology of atherosclerotic cardiovascular diseases (ASCVDs), including coronary artery disease, stroke, and peripheral artery disease. It is defined by Sies as “an imbalance between oxidants and antioxidants in favor of oxidants, leading to disruption of redox signaling and/or molecular damage” [2]. This imbalance arises from the increased production of pro-oxidants and/or impaired antioxidant defense systems [3]. Oxidative stress and inflammation are closely interconnected processes. Pro-oxidants stimulate the production of pro-inflammatory cytokines, while inflammation further amplifies oxidative stress [4,5].

In this context, diet—one of the most important modifiable risk factors—has been shown to directly influence the incidence of ASCVDs [6], partly through its ability to modulate oxidative stress and inflammation [7]. Oxidative damage can be mitigated by endogenous and dietary antioxidants [8,9,10]. In particular, higher dietary intake of certain exogenous antioxidants, such as vitamin C, carotenoids, and α-tocopherol, has been associated with a reduced risk of CVD and all-cause mortality in longitudinal studies [11]. However, intervention trials using single antioxidants or combinations thereof have yielded inconsistent results. In some cases, supplementation has even been associated with adverse outcomes [12,13,14].

These seemingly paradoxical findings may be explained by several factors. First, whole foods contain thousands of bioactive compounds that interact synergistically, an effect that is difficult to replicate with isolated supplements. Second, fat-soluble antioxidants, such as vitamins A and E and β-carotene, carry a greater risk of bioaccumulation and toxicity than water-soluble compounds, such as vitamin C, because they are stored in adipose tissue [15]. Together, these observations suggest that supplementation with isolated antioxidants may fail to reproduce the complex biochemical interactions that occur within whole-food matrices [5].

Given the importance of antioxidant synergy and the multifactorial nature of oxidative stress, which is shaped by both antioxidant and pro-oxidant exposures, composite measures may provide a more comprehensive assessment than individual components. The Oxidative Balance Score (OBS) is a holistic index that captures cumulative exposure to factors influencing oxidative stress across dietary nutrients, and lifestyle domains [16]. A validation study using the EPIC cohort demonstrated that the OBSs integrating nutrient, food, and lifestyle components are valid tools for assessing an individual’s oxidative balance [17].

Associations between OBSs and markers of oxidative stress [17,18,19,20], inflammation [19,20,21], and CVD risk factors, including hypertension [22,23], circulating lipids [20,23], and metabolic syndrome, have been reported in the literature. Although previous studies have compared different OBSs (using, foods, and lifestyle factors as components), evidence remains limited in older adults. Furthermore, relatively few studies have specifically examined the relationship between OBSs and biomarkers of oxidative stress. In this context, the objectives of the present exploratory pilot study were to: (1) compare baseline OBSs among older adults across cardiovascular-risk groups; and (2) examine the associations between these scores and plasma biomarkers of oxidative stress, inflammation, lipid metabolism, and liver-related biomarkers.

## 2. Materials and Methods

### 2.1. Study Population

In this cross-sectional study, a total of 44 participants aged 65–84 years were recruited and stratified into three groups according to cardiovascular risk: (1) healthy individuals free from clinical and subclinical manifestations of atherosclerosis (*n* = 16); (2) individuals with hypercholesterolemia (*n* = 14); and (3) post-myocardial infarction (post-MI) patients (*n* = 14).

Participants were recruited through advertisements displayed in public locations, including shopping malls, clinics, hospital waiting rooms, and retirement homes. Post-MI participants were additionally recruited from the geriatrics and cardiology departments. Inclusion criteria for the healthy group included the absence of a family history of CVD, normal blood pressure (<130/85 mm Hg), a body mass index (BMI) between 23 and 28 kg/m^2^, and a normal electrocardiogram. The hypercholesterolemia group consisted of newly diagnosed, untreated individuals with LDL-cholesterol levels between 3.5 and 5.0 mmol/L; individuals with familial hypercholesterolemia were excluded.

The post-MI group included patients assessed at least 3 months after the index event to allow for the stabilization of inflammatory processes. Exclusion criteria applied to all groups and included diabetes or prediabetes (HbA1c > 6.0%), current smoking, chronic inflammatory conditions (e.g., renal failure, active cancer), gastrointestinal diseases affecting nutrient absorption, and use of antioxidant and omega-3 fatty acid supplements.

This pilot study was conducted in accordance with the principles of the Declaration of Helsinki. The study protocol was approved by the Research Ethics Committee of the Integrated University Health and Social Services Center of Estrie–Sherbrooke University Hospital Center (protocol #: #2019-3145; approval date: 27 May 2020). All participants provided written informed consent prior to enrollment.

### 2.2. Measurements

#### 2.2.1. Sociodemographic and Health Data

Sociodemographic and health data were collected using a standardized questionnaire administered by a trained study nurse. Collected variables included age, sex, occupation, education level, medical history, current medications, physical activity level, tobacco and alcohol use, adherence to specific dietary patterns, and blood pressure. Additional relevant clinical information was recorded. These data were used to characterize the study population and to calculate the OBSs.

#### 2.2.2. Anthropometric Measurements

Body weight and height were measured at baseline by a trained nurse, with participants being barefoot and wearing light clothing, using a calibrated scale and stadiometer. Waist circumference (WC) was measured in centimeters using a non-stretchable, graduated tape measure according to standard procedures. Body mass index (BMI) was calculated as weight (kg) divided by height squared (m^2^) and classified according to the World Health Organization cut-off points [24]. Although BMI is widely used to assess overweight and obesity, WC was considered the primary indicator of adiposity in this study because aging is associated with a progressive decline in lean mass and a concomitant increase in fat mass [25].

#### 2.2.3. Dietary Intake

Dietary intake was assessed once using a 3-day food diary, including two weekdays and one weekend day, completed at recruitment. Each diary was reviewed by the study’s registered dietitian to identify potential errors or missing entries. When necessary, participants were contacted to clarify or complete the recorded information. Dietary data were analyzed using the Nutrific^®^ web application (version 1.1, 2010, Université Laval, Québec, QC, Canada). The software converts the average dietary intake from the 3-day food record into estimates of total energy intake (TEI, kcal/day) and nutrient composition, including macronutrients (proteins, carbohydrates, and fats), micronutrients (vitamins and minerals), and certain phytochemicals.

#### 2.2.4. Calculation of Nutrient-, Food- and Lifestyle-Based Oxidative Balance Scores

Using data from the food diary and the health questionnaire, three individual OBSs and two combined scores were calculated for each participant based on a priori evidence regarding their association with oxidative stress [16,17]. The nutrient-OBS (OBSN), food-OBS (OBSF), and lifestyle-OBS (OBSL) comprised 15 dietary components, 9 food components, and 2 lifestyle components, respectively. Antioxidant components included vitamins A, C, E, and B9; β-carotene, lutein/zeaxanthin, lycopene, monounsaturated fatty acids, selenium, zinc, fiber, and calcium, as well as food groups, such as fruits, vegetables, legumes, fish, and nuts, in addition to physical activity. Pro-oxidant components included iron, saturated fatty acids, polyunsaturated fatty acids, and food groups such as red and processed meats, refined grains and cereals, sweets, salty snacks, and alcohol. Physical activity was categorized, according to Health Canada’s physical activity level (NAP) classifications, as inactive, moderately active, or active [26]. The rationale for the inclusion of antioxidant and pro-oxidant components in OBSs has been described previously [16]. Dietary components were adjusted for total energy intake. Two additional composite scores were derived: the lifestyle–nutrient-based OBS (nutrient–lifestyle OBS) (OBSN-L) and the lifestyle–food-based OBS (food–lifestyle OBS) (OBSF-L) comprising 17 and 11 components, respectively.

All continuous and categorical variables were categorized into tertiles based on their distribution within the study sample. For antioxidant components, which are presumed to mitigate oxidative stress, participants in the lowest tertile 1 (T1) were assigned 0 points; those in the intermediate tertile (T2) were assigned 1 point; and those in the highest tertile (T3) were assigned 2 points. Conversely, for pro-oxidant components, which are considered to promote oxidative stress, participants in T1 were assigned 2 points; those in T2 were assigned 1 point; and those in T3 were assigned 0 points. A higher OBS reflects a more favorable oxidative balance, characterized by greater exposure to antioxidant than pro-oxidant factors. The theoretical score ranges for the five oxidative balance scores were as follows: 0 to 30 for OBSN; 0 to 18 for OBSF; 0 to 4 for OBSL; 0 to 34 for OBSN-L; and 0 to 22 for OBSF-L. Detailed information on the score components and point allocation is provided in Supplementary Table S1.

### 2.3. Biomarkers

Fasting blood samples were collected at baseline after a minimum of 8 h of fasting in EDTA tubes. Samples were immediately centrifuged (4000 rpm for 15 min), and plasma was aliquoted and stored at −80 °C until analysis. The following biomarkers were assessed: (a) oxidative stress markers, including Trolox Equivalent Antioxidant Capacity (TEAC) and Ferric Reducing Antioxidant Power (FRAP); (b) the inflammatory marker, C-reactive protein (CRP); and (c) lipid metabolism and liver-related biomarkers, including high-density lipoprotein (HDL)-cholesterol, alanine aminotransferase (ALT), and aspartate aminotransferase (AST).

TEAC and FRAP were measured in plasma using commercial assay kits—the TEAC assay kit (ABTS; Cell Biolabs, Inc., San Diego, CA, USA) and the FRAP Assay Kit (MAK509, Sigma-Aldrich, St. Louis, MO, USA)—according to the manufacturer’s instructions. The remaining biomarkers were analyzed at the Clinical Biochemistry Laboratory of the Sherbrooke University Hospital Center using standardized automated methods. Plasma CRP was quantified using an immunoassay based on antigen–antibody reactions. HDL cholesterol, ALT, and AST concentrations were determined using enzymatic assays.

### 2.4. Statistical Analysis

All analyses were performed using IBM SPSS Statistics (IBM Corporation, Armond, NY, USA, version 29, 2025), with statistical significance set at *p* < 0.05. Data normality was assessed using the Shapiro–Wilk test, complemented by the evaluation of skewness (z-score) and kurtosis indices. Bootstrapping procedures were applied to obtain robust estimates and confidence intervals without relying on normality assumptions.

To compare the three groups, a one-way analysis of variance (ANOVA) was used for continuous variables with a normal distribution. Homogeneity of variances was assessed using Levene’s test; when this assumption was violated, Welch’s ANOVA was applied instead. When overall group differences were significant, Bonferroni post hoc tests were conducted to identify pairwise differences. For non-normally distributed variables, the Kruskal–Wallis test was used. Categorical variables were analyzed using Fisher’s exact test, as all variables included at least one expected frequency below five. When significant differences were observed, Bonferroni-adjusted post hoc comparisons were performed. Correlations between OBSs and biomarkers were assessed using Pearson correlation coefficients for normally distributed variables (TEAC, FRAP, HDL, AST) and Spearman rank correlation coefficients for non-normally distributed variables (CRP, ALT). Results were visualized using a heatmap.

To further examine the association between OBSs (independent variables) and biomarkers (dependent variables), two linear regression models were constructed. Model 1 included age, sex, and clinical subgroup only. Model 2 additionally included the OBS variable of interest, while retaining age, sex, and clinical subgroup as covariates. Variables were entered simultaneously using the Enter method. Regression assumptions, including linearity, homoscedasticity, normality of residuals, and absence of multicollinearity, were verified. No evidence of problematic multicollinearity or highly influential observations was detected; VIF values ranged from 1.001 to 1.047, tolerance values ranged from 0.808 to 0.999, and all Cook’s distances were <1 (Supplementary Table S2). The tertile cut-offs were derived from the full study sample.

Age and sex were selected because they are well-established determinants of CVD risk. The clinical subgroup was included because the post-MI group received therapies with potentially different effects on lipid and liver biomarkers. To avoid overadjustment, alcohol intake and physical activity were intentionally not included as additional covariates because they are components of the lifestyle-integrated OBS scores. Because of the non-normal distribution of several variables, bootstrap methods were used for all regression analyses. Regression coefficients, standard errors, bias-corrected and accelerated (BCa) 95% confidence intervals, and *p*-values were estimated from 5000 bootstrap resamples. Statistical significance was determined based on *p* < 0.05, while BCa 95% CIs were reported as complementary measures of estimation uncertainty.

The primary analyses were prespecified to address the study objectives, namely (1) to compare baseline OBSs across clinical groups; and (2) to examine the associations between OBSs and biomarkers of oxidative stress, inflammation, and lipid metabolism and liver-related biomarkers. Evaluating the contribution of individual OBS components was considered an exploratory analysis. No correction for multiple comparisons was applied to the component-level analyses; therefore, the corresponding *p*-values are nominal, and these findings should be considered exploratory and hypothesis-generating.

## 3. Results

### 3.1. Characteristics of Participants by Cardiovascular Risk Group

A total of 44 older adults with varying levels of cardiovascular risk were included in the analysis, comprising 16 healthy individuals, 14 participants with hypercholesterolemia, and 14 post-myocardial infarction (post-MI) participants. Demographic and clinical characteristics of the study population stratified by clinical subgroup are presented in Table 1.

The healthy group showed a balanced sex distribution, whereas the hypercholesterolemia group had a higher proportion of women (78.6%) and the post-MI group had a lower proportion (21.4%) (*p* = 0.008). As expected, medication use was more prevalent in the post-MI (100%) and hypercholesterolemia (50%) groups than in healthy participants (25%) (*p* < 0.001). Post-MI participants were receiving pharmacological treatment, most commonly statins, antiplatelet agents, β-blockers, and ACE inhibitors/ARBs. Regarding biochemical parameters, HDL cholesterol levels were significantly lower in the post-MI group (1.19 ± 0.26) than in the healthy (1.69 ± 0.50) and hypercholesterolemia (2.00 ± 0.50) groups (*p* < 0.001). In contrast, ALT and AST levels were significantly higher in the post-MI group (31.15 ± 15.56 and 27.46 ± 10.18, respectively) than hypercholesterolemia (15.45 ± 3.17 and 21.27 ± 5.71) and healthy groups (17.79 ± 5.26 and 20.64 ± 5.03) (*p* < 0.001 and *p* = 0.034, respectively). Post hoc analysis revealed statistically significant differences between the post-MI group and both the hypercholesterolemia and healthy groups, whereas no significant differences were observed between the healthy and hypercholesterolemia groups.

Age, years of education, anthropometric measures, alcohol consumption, physical activity levels, and the remaining biomarkers (TEAC, FRAP, and CRP) did not differ significantly across groups (all *p* > 0.05).

### 3.2. Nutrient Intake by Cardiovascular Risk Group

Dietary intake is presented in Table 2. Total energy intake did not differ significantly between groups (*p* > 0.05). The macronutrient distribution (percentage of total energy from carbohydrates, fats, and proteins) was comparable across clinical subgroups. Although not statistically significant, the proportion of energy derived from monounsaturated fatty acids (MUFA) was lower in post-MI participants (12.07 ± 3.72%) than in the hypercholesterolemia (16.83 ± 6.19%) and healthy (16.83 ± 7.07%) groups.

Regarding micronutrient intake, differences were observed for selected antioxidant-related nutrients. Post hoc analysis showed that vitamin A intake, expressed as a percentage of the recommended dietary allowance (RDA), was significantly lower in the post-MI group (80.13 ± 34.09%) than in the hypercholesterolemia group (119.13 ± 58.11%) (*p* = 0.047). Similarly, zinc intake (% RDA) was significantly lower in the post-MI group (81.82 ± 32.98%) than in the healthy group (115.47 ± 41.03%) (*p* = 0.045). Other micronutrients, including vitamin C, vitamin E, and selenium, did not differ significantly between groups (*p* > 0.05).

### 3.3. Food Group Consumption by Cardiovascular Risk Group

Food group consumption is summarized in Table 3. Post hoc analysis showed that healthy participants reported significantly higher red meat intake (62.51 ± 50.39 g) than both hypercholesterolemia (26.36 ± 41.02 g) and post-MI groups (11.54 ± 22.99 g; *p* = 0.007). Processed meat intake tended to be higher in the post–MI (25.79 ± 37.69 g) and hypercholesterolemia (22.64 ± 26.53 g) groups than in healthy participants (7.23 ± 9.55 g), although these differences were not statistically significant. Conversely, vegetable intake tended to be lower in the post-MI (203.04 ± 152.35 g) and hypercholesterolemia (203.05 ± 117.08 g) groups than in the healthy group (305.20 ± 188.18 g). No significant differences were observed for fat, fruits, cereals, legumes, nuts and seeds, fish/seafood, poultry, dairy products, or total meat intake (*p* > 0.05).

### 3.4. Oxidative Balance Scores

Among the five OBSs evaluated, only OBSN and OBSN–L differed significantly across the three clinical subgroups (*p* = 0.005 and *p* = 0.010, respectively) (Table 4). Participants with hypercholesterolemia exhibited the highest OBS values, followed by healthy individuals, whereas the post-MI group had the lowest scores. Post hoc analyses indicated that OBSN was significantly lower in post-MI participants (12.50 ± 4.43) than in participants with hypercholesterolemia (18.00 ± 4.30; *p* = 0.005). No significant differences were observed between healthy participants and the other groups. Similar patterns were observed for OBSN–L. No significant differences were found for OBSF, OBSL, and OBSF–L.

### 3.5. Correlations Between Oxidative Balance Scores and Biomarkers

**Figure 1:**
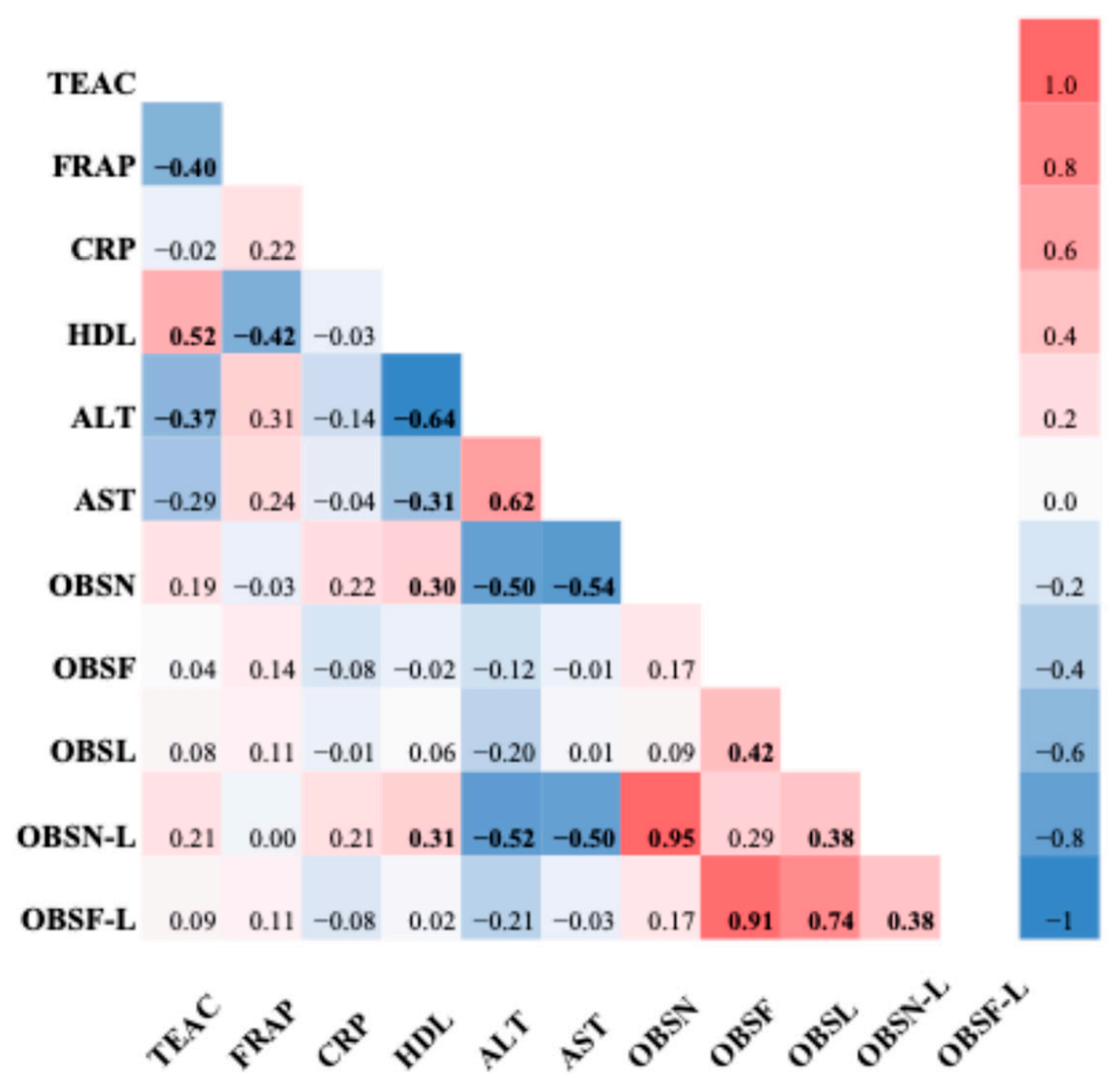
Correlation matrix between dietary scores (OBSN, OBSF, OBSL, OBSN–L and OBSF–L) and biomarkers of oxidative stress, inflammation, lipid metabolism and liver-related biomarkers in older adults with different cardiovascular risk.

Bivariate correlations between OBSs and circulating biomarkers of oxidative stress, inflammation, and lipid metabolism and liver-related function are presented in Figure 1. Higher OBSN and OBSN–L were positively associated with HDL cholesterol (r = 0.302, *p* = 0.046 and r = 0.305, *p* = 0.046, respectively). In contrast, OBSN and OBSN–L were inversely correlated with ALT (r = −0.496, *p* = 0.001 and r = −0.519, *p* <0.001, respectively) and AST (r = −0.537, *p* < 0.001 and r = −0.503, *p* = 0.001, respectively). No significant correlations were observed between OBSs and biomarkers of oxidative stress (TEAC and FRAP) or inflammation (CRP) (all *p* > 0.05).

Heatmap color intensity represents the strength and direction of the correlation coefficients, ranging from blue (negative correlations) to red (positive correlations), with white indicating no correlation. Pearson and Spearman correlation coefficients were calculated for normally distributed and non-normally distributed variables, respectively. Correlations in bold were all statistically significant (*p* < 0.05). Abbreviations: TEAC, trolox equivalent antioxidant capacity; FRAP, ferric reducing antioxidant power; CRP, C-reactive protein; HDL, high-density lipoprotein; ALT, alanine aminotransferase; AST, aspartate aminotransferase.

### 3.6. Linear Association Between Oxidative Balance Scores and Biomarkers

Results of linear regression analyses examining the association between OBSN, OBSN–L, and OBSL, and the circulating biomarkers are shown in Table 5.

Higher OBSN was significantly associated with AST levels in the adjusted model. After adjustment for age, sex, and clinical group, the associations remained statistically significant (per one-point increase in OBS: β = −0.64; *p* = 0.022 for AST). Similarly, after adjustment for confounders, higher OBSN–L was significantly associated with ALT and AST levels (per one-point increase in OBSN–L: β = −1.06; *p* = 0.048, and β = −0.54; *p* = 0.034, respectively).

No significant associations were observed for OBSF, OBSF–L, or OBSL with any biomarkers. In addition, none of the OBS measures were significantly associated with TEAC, FRAP, or CRP. 

### 3.7. Linear Association Between Individual Nutrient and Lifestyle Components and Biomarkers

Results of the linear regression analyses examining the association between individual OBS components and biomarkers are presented in Table 6.

Multiple regression analyses, adjusted for age, sex, and clinical group, showed that several OBS components were significantly associated with AST and ALT levels. Specifically, vitamin C and vitamin A were associated with AST (β = −2.96 and −3.69, respectively; all *p* < 0.05). Vitamin C and fiber were significantly associated with ALT levels (β = −4.11 and −4.17, respectively; all *p* < 0.05). No significant associations were observed between individual OBS components and HDL cholesterol levels.

## 4. Discussion

This pilot exploratory study provides novel contributions to the OBS research field in several ways. First, it suggests that dietary oxidative balance—particularly OBSN–L—is independently associated with more favorable hepatic biomarker profiles in older adults across different cardiovascular-risk groups. Higher OBSN–L was associated with lower ALT and AST concentrations; these associations persisted after adjustment for age, sex, and clinical group. Second, this study evaluated multiple OBS constructs in a cohort of older adults across different levels of cardiovascular risk, including post-MI patients. Third, the simultaneous assessment of oxidative stress, inflammatory, lipid metabolism and liver-related biomarkers provide a more comprehensive evaluation of the biological relevance of OBSs. Because of the cross-sectional design, the observed associations cannot be interpreted as evidence of causal relationships or underlying biological mechanisms. Reverse causation and residual confounding cannot be excluded. Consequently, these findings should be considered hypothesis-generating and require confirmation in larger prospective studies.

Our findings also contribute to the limited amount of studies in the literature examining the relationship between oxidative balance and liver function. Zhou et al. [26] reported that a dietary and lifestyle-based OBS was associated with liver enzymes, demonstrating inverse associations with ALP but positive associations with ALT and AST. They further showed that dietary components primarily drove the associations [26]. While Zhou et al. reported inconsistent relationships between OBS and individual liver enzymes, both their findings and ours support the hypothesis that oxidative balance is related to hepatic function.

The association between OBSs and liver enzyme concentrations could partly be explained by the concept of “parahormesis”, whereby phytochemical antioxidants stimulate endogenous antioxidant defense systems and activate cellular stress-response pathways in the liver [27,28], rather than acting solely as direct radical scavengers in the blood. Many dietary polyphenols reach the colon intact and are subsequently metabolized by the gut microbiota into bioactive secondary metabolites capable of modulating hepatic and systemic redox pathways [29]. In addition, metabolic liver disease and ASCVD share common pathophysiological mechanisms, including oxidative stress, systemic inflammation, gut microbiota alterations, endothelial dysfunction, and insulin resistance [28,30].

Our study does not provide evidence that the calculated OBSs are associated with HDL cholesterol in older adults. Previous studies have reported inverse associations between OBSs and low HDL cholesterol, with potential sex-specific effects [22,23]. Specifically, participants in the highest OBS category exhibited 52% lower odds of having low HDL cholesterol in women but 63% higher odds in men compared with those in the lowest category. Given the role of estrogen in regulating triglyceride and cholesterol metabolism, sex-related differences in lipid metabolism may partly explain these findings [31].

Most studies report strong inverse associations between OBS and biomarkers of oxidative stress [18,19] and inflammation [20,21,22]. In contrast, we did not observe significant associations between OBSs and plasma TEAC or CRP. The lack of associations in our study may reflect differences in sample size, OBS construction (dietary vs. biomarker-based), and population characteristics, including the inclusion of medicated post-MI patients. It may also reflect the limited ability of TEAC assays to accurately capture oxidative stress in vivo. Although plasma TEAC can be useful for evaluating dietary responses when used alongside other biomarkers [32], it is not considered a reliable standalone indicator of oxidative stress status in vivo. Moreover, TEAC is an indirect marker of circulating antioxidant capacity and does not provide a comprehensive assessment of oxidative stress. It is influenced by circulating compounds, such as uric acid, and may not directly represent oxidative injury to lipids, proteins, or DNA. In contrast, oxidized LDL, protein carbonyls, DNA oxidation markers, and particularly F2-isoprostanes are regarded as more robust and specific measures of oxidative damage [33]. Other possible explanations include limited statistical power, limitations of the OBS construct, including dietary measurement error, clinical heterogeneity, and the possibility that OBSs do not reflect systemic oxidative status in this population.

Our findings suggest that post-MI patients had lower dietary antioxidant intake reflected by lower overall OBSN at baseline, despite having similar or even a tendency toward higher OBSF scores compared with healthy and hypercholesterolemia patients. Although post-MI patients appeared to reduce red meat intake (a major pro-oxidant exposure), they did not achieve adequate intake of several key antioxidant nutrients, including vitamin A and zinc. In addition, post-MI participants tended to consume fewer vegetables and more processed meat than the other groups. However, the OBSF did not reproduce the associations observed with the nutrient-based scores (OBSN and OBSN–L). This discrepancy underscores that oxidative balance may depend more on cumulative nutrient exposure than on food group classification.

Moreover, food-based assessments cannot fully account for exposure to environmental contaminants (e.g., pesticides, heavy metals, preservatives, additives, and neo formed compounds) [34], as well as the large number of food contact chemicals that may influence metabolic outcomes [35]. In addition, most existing OBS frameworks integrate both diet and lifestyle components, rather than isolating food-based contributions. Although one study reported that higher OBSF–L was associated with lower CRP levels, this relationship appeared to be driven primarily by lifestyle factors rather than dietary components [17]. These findings suggest that simply reducing pro-oxidant food intake may be insufficient if antioxidant nutrient density is not simultaneously optimized. Taken together, these observations support further investigation of nutrient-focused dietary approaches in secondary cardiovascular prevention.

Interestingly, our exploratory analyses showed that three antioxidant nutrient components—vitamin C, vitamin A, and fiber—were associated with hepatic biomarkers (ALT and AST). Vitamin C is widely recognized as a potent and relatively non-toxic antioxidant due to its ability to scavenge free radicals, such as hydroxyl, peroxyl, and superoxide radicals, in aqueous environments [34]. Vitamin A, a fat-soluble antioxidant, may also contribute to reducing oxidative stress in hepatic cells. It functions as a chain-breaking antioxidant by neutralizing peroxyl radicals before they initiate the propagation phase of lipid peroxidation, thereby preventing the formation of lipid hydroperoxides [33]. The liver is susceptible to oxidative stress because of its central role in lipid metabolism and its high mitochondrial activity. Excessive oxidative stress in hepatocytes may lead to the release of ALT and AST into the circulation. These enzymes are widely used as indicators of hepatocellular injury and may indirectly reflect oxidative stress damage-related liver damage [36,37].

A review of the literature indicates that fiber is rarely included as an individual component of OBS frameworks [16], despite its potential indirect antioxidant effects. First, dietary fiber reaches the colon, where it is fermented by the gut microbiota into short-chain fatty Acids (SCFAs). These metabolites are absorbed by enterocytes and hepatocytes, enter the systemic circulation, and exert metabolic effects [37]. Notably, SCFAs can activate the Nrf2 pathway, enhancing endogenous antioxidant defenses [38]. Second, fiber can act as a carrier of polyphenols, forming «antioxidant dietary fiber» complexes. These complexes may exhibit greater antioxidant capacity when released from colonic fermentation compared with digestion into small intestine [37]. Additional mechanisms, such as metal chelation and improved glycemic control, may further contribute to fiber’s antioxidant role [39].

Collectively, these exploratory findings suggest that the association between nutrient-based OBSs and hepatic biomarkers may reflect the combined influence of multiple antioxidant nutrients acting through complementary biological pathways rather than the effect of any single dietary component. Such an approach provides a more comprehensive and robust representation of oxidative balance.

This observation supports the use of composite dietary oxidative balance scores, which are designed to capture the synergistic nature of dietary exposures. However, given the exploratory nature of the component-level analyses and the cross-sectional design, these findings require confirmation in larger prospective studies.

A major strength of this analysis is the simultaneous evaluation of nutrient-based and food-based oxidative balance scores, including their lifestyle-integrated versions, which enabled a direct comparison of different score constructs. By integrating lifestyle factors alongside dietary components, these scores provide a more comprehensive assessment of the overall oxidative environment. Dietary intake was assessed using three-day food records rather than dietary recall methods, thereby reducing memory bias in this older population. In addition, the inclusion of multiple circulating biomarkers allowed for a multidimensional assessment of lipid metabolism, liver-related biomarkers, oxidative status, and inflammation. Finally, the use of bootstrap confidence intervals in linear regression models strengthened statistical inference without requiring the transformation of clinically interpretable variables.

The main limitation of this pilot study is its modest sample size, which limits statistical power and generalizability. Because of the exploratory nature of this study and the large number of analyses performed, an a priori sample size calculation was not conducted. All post-MI participants were on stable statin therapy (Table S3) prior to the study entry. Because of the modest sample size, adjustment for individual medication classes was not feasible. This represents an important limitation, as medication use differed substantially across the clinical groups, and different drug classes may have influenced the investigated biomarkers through distinct mechanisms. Future studies should include larger sample sizes to allow adjustment for individual medication classes and to confirm these findings in more diverse populations.

Our OBS calculations relied on many dietary components and dietary intake was assessed using three-day food records. Because of intra-individual variability in dietary intake, some degree of exposure misclassification cannot be excluded. Although dietary food records reduce recall bias, they may not adequately represent habitual dietary intake, particularly for episodically consumed foods and micronutrients. Furthermore, information on dietary modifications after myocardial infarction and prior nutritional counselling was not available. Consequently, we cannot exclude the possibility that post-diagnosis lifestyle changes influenced the observed associations. Finally, the equal weighting of OBS components does not account for differences in redox potency of individual antioxidants. Moreover, OBSs do not capture endogenous antioxidant systems, which contribute substantially to overall plasma antioxidant capacity.

Although regression models were adjusted for several key covariates, residual confounding from unmeasured environmental or lifestyle factors cannot be excluded. Furthermore, the modest sample size and cross-sectional design limit statistical power, causal inference, and generalizability. Therefore, these findings should be considered exploratory and require confirmation in larger, well-powered prospective studies before their clinical utility can be established.

## 5. Conclusions

In conclusion, this pilot study suggests that, among different OBS constructs integrating nutrient, food, and lifestyle dimensions, nutrient-based OBSs, with and without lifestyle integration, were more strongly associated with liver-related biomarkers than food-based OBSs in older adults across cardiovascular-risk groups. Exploratory analyses further suggested that individual nutrients, including vitamin C, vitamin A, and fiber, may have contributed to these associations. In contrast, no association was observed between OBSs and circulating total antioxidant capacity or CRP, suggesting that OBSs may reflect broader dietary and lifestyle patterns linked to liver-related biomarkers rather than systemic oxidative status alone. Although the findings should be interpreted cautiously because of the pilot, cross-sectional design, they support the potential relevance of nutrient-based OBSs as integrative tools for characterizing oxidative balance in older adults. Larger prospective studies are needed to confirm these associations and clarify the clinical utility of OBSs in cardiovascular prevention strategies.

## Figures and Tables

**Table 1:** Descriptive characteristics of study participants.

Characteristics	Healthy (*n* = 16) ^(a)^	Hyperlipidemic (*n* = 14) ^(a)^	Post-MI (*n* = 14) ^(a)^	*p*-Value ^(b)^	η^2 (c)^
Age (years)	73.86 ± 5.31	73.50 ± 5.26	73.89 ± 6.17	0.985	0.001
Education (years)	15.36 ± 3.80	14.38 ± 2.92	13.56 ± 3.68	0.071	0.127
Gender				**0.008**	--
Females	7 (43.8)	11 (78.6)	3 (21.4)		
Males	9 (56.3)	3 (21.4)	11 (78.6)		
Weight (kg)	73.49 ± 17.61	66.59 ± 12.60	77.36 ± 12.60	0.206	0.089
Height (cm)	166.07 ± 8.91	159.94 ± 8.88	170.11 ± 10.90	0.132	0.094
BMI (kg/m^2^)	27.05 ± 4.83	26.00 ± 3.72	26.96 ± 3.34	0.789	0.058
Waist circumference (cm)	106.59 ± 26.15	91.91 ± 11.36	99.07 ± 10.97	0.125	0.101
Medication use				**<0.001**	--
Yes	4 (25)	8 (58)	14 (100)		
No	12 (75)	6 (42)	0 (0)		
Alcohol (weekly consumption)	3.39 ± 2.81	7.16 ± 6.60	7.15 ± 9.00	0.249	0.053
Physical activity (min/week)	178.6 ± 142.4	207.3 ± 206.4	140.8 ± 163.3	0.632	0.097
TEAC (µM TE)	1510.1 ± 211.1	1575.2 ± 77.64	1486.2 ± 216.4	0.475	0.046
FRAP (µM Fe^2+^)	863.49 ± 153.8	750.69 ± 111.4	803.09 ± 123.0	0.082	0.145
CRP (mg/L)	1.67 ± 1.19	2.21 ± 1.65	1.28 ± 0.68	0.184	0.001
HDL (mmol/L)	1.69 ± 0.50 ^a^	2.00 ± 0.50 ^ab^	1.19 ± 0.26 ^c^	**<0.001**	0.359
ALT (UI/L)	17.79 ± 5.26 ^a^	15.45 ± 3.17 ^ab^	31.15 ± 15.56 ^c^	**<0.001**	0.333
AST (UI/L)	20.64 ± 5.03 ^a^	21.27 ± 5.71 ^ab^	27.46 ± 10.18 ^c^	**0.034**	0.155

Data are expressed as mean ± standard deviation for continuous variables and as frequency (percentage) for categorical variables. Abbreviations: BMI, body mass index; TEAC, Trolox equivalent antioxidant capacity; CRP, C-reactive protein; HDL, high density lipoprotein; ALT, alanine aminotransferase; AST, aspartate aminotransferase. ^(a)^ Healthy participants (no history of CVD), hyperlipidemic participants, and participants with history of myocardial infarction. ^(b)^ One-way ANOVA with Bonferroni post-hoc test (continuous variables) or Fisher’s exact test (categorical variables). Groups not sharing a common letter differ significantly (*p* < 0.05). Bold indicates statistical significance at *p* < 0.05. ^(c)^ Eta-squared (η^2^).

**Table 2:** Dietary intake (energy, macro- and micronutrient) of study participants.

Energy/Nutrients	Healthy (*n* = 16) ^(a)^	Hyperlipidemic (*n* = 14) ^(a)^	Post-MI (*n* = 14) ^(a)^	*p*-Value ^(b)^	η^2 (c)^
Energy (kcal/d)	1851.9 ± 400.90	1988.0 ± 400.62	1809.60 ± 502.70	0.458	0.037
%RDA	99.44 ± 23.72	109.08 ± 26.41	93.09 ± 17.49	0.214	0.073
Protein (g/d)	84.51 ± 31.71	77.44 ± 17.23	74.92 ± 17.45	0.180	0.080
% E	17.96 ± 5.61	16.77 ± 3.43	15.79 ± 2.90	0.106	0.104
Carbohydrates (g/d)	221.39 ± 37.64	220.12 ± 53.72	216.64 ± 83.42	0.870	0.007
% E	43.00 ± 9.50	48.60 ± 15.61	45.47 ± 14.39	0.786	0.012
Total fat (g/d)	75.42 ± 20.31	84.26 ± 22.95	70.45 ± 24.40	0.289	0.059
% E	36.63 ± 11.66	41.59 ± 12.24	32.72 ± 7.55	0.123	0.097
SFA (g/d)	24.29 ± 7.00	25.83 ± 9.90	23.92 ± 10.13	0.785	0.012
% E	11.74 ± 3.89	12.62 ± 4.59	11.18 ± 4.32	0.605	0.024
MUFA (g/d)	34.25 ± 13.45	33.87 ± 10.90	26.15 ± 11.29	0.138	0.092
% E	16.83 ± 7.07	16.83 ± 6.19	12.07 ± 3.72	0.059	0.129
PUFA (g/d)	13.94 ± 5.68	16.26 ± 6.42	14.24 ± 5.41	0.696	0.018
% E	6.68 ± 3.5	8.02 ± 3.17	6.64 ± 1.87	0.472	0.036
Trans fat (g/d)	1.05 ± 0.53	1.17 ± 0.83	1.17 ± 1.12	0.889	0.006
% E	0.51 ± 0.28	0.57 ± 0.40	0.56 ± 0.55	0.900	0.005
Fibres (g/d)	20.04 ± 5.10	21.96 ± 6.90	21.64 ± 15.58	0.961	0.002
% AI	81.10 ± 27.99	97.86 ± 35.37	76.95 ± 51.91	0.403	0.043
Vitamin C (%RDA)	169.70 ± 94.63	175.26 ± 107.45	107.20 ± 50.45	0.072	0.120
Vitamin E (%RDA)	51.47 ± 12.19	63.08 ± 20.18	45.73 ± 29.20	0.109	0.102
Vitamin A (%RDA)	85.02 ± 33.10 ^ab^	119.13 ± 58.11 ^a^	80.13 ± 34.09 ^b^	**0.047**	0.138
Selenium (%RDA)	241.87 ± 140.54	251.07 ± 196.17	175.84 ± 55.03	0.301	0.057
Zinc (%RDA)	115.47 ± 41.03 ^a^	108.70 ± 27.88 ^ab^	81.82 ± 32.98 ^b^	**0.045**	0.140
β-carotene (μg/d)	4121 ± 2439	5729 ± 3888	3399 ± 2902	0.139	0.047
Lutein/zeaxanthin (μg/d)	2327 ± 1932	2140 ± 1290	2101 ± 2332	0.941	0.003
Lycopene (μg/d)	3701 ± 5324	5394 ± 7849	1218 ± 1784	0.148	0.089

Data are expressed as mean ± standard deviation for continuous variables. Abbreviations: % E, percentage of total energy intake; AI, adequate intake, RDA, recommended dietary allowances; SFA, saturated fatty acid; MUFA, monounsaturated fatty acids; PUFA, polyunsaturated fatty acids. ^(a)^ Healthy participants (no history of CVD), hyperlipidemic participants, and participants with history of myocardial infarction. ^(b)^ One-way ANOVA with Bonferroni post-hoc test (continuous variables). Groups not sharing a common letter differ significantly (*p* < 0.05). Bold indicates statistical significance at *p* < 0.05. ^(c)^ Eta-squared (η^2^).

**Table 3:** Food groups consumption of study participants.

Food Groups	Healthy (*n* = 16) ^(a)^	Hyperlipidemic (*n* = 14) ^(a)^	Post-MI (*n* = 14) ^(a)^	*p*-Value ^(b)^	η^2 (c)^
Fats (g/d)	20.11 ± 15.41	13.72 ± 13.44	10.36 ± 12.29	0.151	0.088
Vegetables (g/d)	305.20 ± 188.18	203.05 ± 117.08	203.04 ± 152.35	0.134	0.093
Fruits (g/d)	214.61 ± 115.53	267.31 ± 186.21	247.32 ± 117.80	0.458	0.037
Cereals (including bread and potatoes) (g/d)	66.33 ± 57.13	63.72 ± 96.37	23.16 ± 30.69	0.148	0.089
Legumes (g/d)	27.93 ± 35.25	15.82 ± 20.61	24.27 ± 39.27	0.650	0.021
Nuts and seeds (g/d)	3.35 ± 7.02	9.87 ± 10.48	7.31 ± 16.66	0.299	0.057
Fish and seafood (g/d)	36.43 ± 55.28	38.63 ± 53.51	57.33 ± 56.82	0.506	0.033
Poultry (g/d)	31.38 ± 50.06	23.81 ± 25.02	17.48 ± 21.53	0.483	0.035
Milk and dairy products (g/d)	168.73 ± 137.31	204.84 ± 147.16	207.71 ± 155.97	0.729	0.015
Red Meat (g/d)	62.51 ± 50.39 ^a^	26.36 ± 41.02 ^b^	11.54 ± 22.99 ^b^	**0.007**	0.248
Processed meat (g/d)	7.23 ± 9.55	22.64 ± 26.53	25.79 ± 37.69	0.119	0.099
Meat and processed meat (g/d)	86.05 ± 80.28	46.45 ± 37.33	38.48 ± 48.22	0.098	0.107
Alcohol (g/d)	6.02 ± 7.56	12.06 ± 13.42	7.63 ± 12.42	0.659	0.002

Data are expressed as mean ± standard deviation for continuous variables. ^(a)^ Healthy participants (no history of CVD), hyperlipidemic participants, participants with a history of myocardial infarction. ^(b)^ One-way ANOVA with Bonferroni post-hoc test (continuous variables). Groups not sharing a common letter differ significantly (*p* < 0.05). Bold indicates statistical significance at *p* < 0.05. ^(c)^ Eta-squared (η^2^).

**Table 4:** Oxidative balance scores of study participants.

OBS	Healthy (*n* = 16) ^(a)^	Hyperlipidemic (*n* = 14) ^(a)^	Post-MI (*n* = 14) ^(a)^	*p*-Value ^(b)^	η^2 (c)^
OBSN	16.25 ± 4.15 ^ab^	18.00 ± 4.30 ^a^	12.50 ± 4.43 ^b^	**0.005**	0.227
OBSF	8.63 ± 1.45	8.50 ± 3.61	9.79 ± 2.78	0.387	0.045
OBSL	2.13 ± 1.31	2.57 ± 1.83	2.43 ± 1.40	0.713	0.016
OBSN-L	18.34 ± 4.72 ^ab^	20.57 ± 4.42 ^a^	14.93 ± 4.97 ^b^	**0.010**	0.200
OBSF-L	10.75 ± 2.05	11.07 ± 4.80	12.21 ± 3.81	0.531	0.030

Data are expressed as mean ± standard deviation for continuous variables and as frequency (percentage) for categorical variables. ^(a)^ Healthy participants (no history of CVD), hyperlipidemic participants, participants with a history of myocardial infarction. ^(b)^ One-way ANOVA with Bonferroni post-hoc test (continuous variables). Groups not sharing a common letter differ significantly (*p* < 0.05). Bold indicates statistical significance at *p* < 0.05. ^(c)^ Eta-squared (η^2^).

**Table 5:** Coefficients of multiple linear regression models examining the association between nutrient-based OBS and lifestyle nutrient-based OBS and circulating biomarkers (N = 44).

	Model 1	Model 2			
	R^2^	β (95% CI)	β (SE)	*p* Value	R^2^
OBSN					
HDL	0.375	0.017 (−0.012, 0.051)	0.017	0.291	0.377
ALT	0.223	−1.081 (−2.042, −0.180)	0.455	0.065	0.390
AST	0.177	−0.639 (−1.092, −0.123)	0.255	**0.022**	0.304
OBSN-L					
HDL	0.375	0.017 (−0.007, 0.045)	0.014	0.243	0.382
ALT	0.223	−1.064 (−1.968, −0.167)	0.411	**0.048**	0.426
AST	0.177	−0.544 (−0.985, −0.044)	0.240	**0.034**	0.290

The regression coefficients (β), the corresponding 95% confidence intervals (CI) and standard errors (SEs), and adjusted R^2^ are shown (representing the proportion of variance explained). Bold indicates statistical significance at *p* < 0.05. Abbreviations: HDL, High-density lipoprotein cholesterol; ALT, alanine aminotransferase; AST, aspartate aminotransferase. Model 1 included age, sex, and clinical subgroup only. Model 2 additionally included the OBS variable of interest.

**Table 6:** Coefficients of multiple linear regression models examining the association between individual nutrient and lifestyle components and circulating biomarkers (N = 44).

	AST				ALT			
	β (95% CI)	β (SE)	*p* Value	R^2^	β (95% CI)	β (SE)	*p* Value	R^2^
Antioxidants								
Vitamin E	−1.031 (−3.532, 1.002)	1.218	0.399	0.167	−3.842 (−8.156, −0.128)	1.800	0.085	0.276
Vitamin C	−2.961 (−5.777, −0.386)	1.365	**0.048**	0.254	−4.109 (−8.370, −0.602)	1.768	**0.049**	0.286
Vitamin A	−3.690 (−6.029, −0.844)	1.200	**0.011**	0.328	−4.386 (−8.680, −0.973)	1.891	0.066	0.309
Lycopene	0.920 (−1.380, 3.403)	1.266	0.465	0.165	−0.845 (−4.814, 3.074)	1.730	0.622	0.207
Lutein /zeaxanthin	−1.939 (−4.580, 0.509)	1.321	0.165	0.202	−2.559 (−6.822, 1.196)	1.826	0.229	0.239
ß-carotene	−2.398 (−4.950, 0.297)	1.285	0.076	0.224	−2.898 (−7.381, 0.955)	2.017	0.192	0.247
Selenium	−2.303 (−5.478, 0.186)	1.367	0.094	0.224	−2.437 (−6.797, 0.974)	1.989	0.256	0.236
Zinc	0.810 (−1.576, 2.891)	1.179	0.492	0.163	−0.936 (−4.809, 2.361)	1.723	0.593	0.207
Fibre	−2.079 (−4.924, 0.519)	1.345	0.129	0.205	−4.171 (−7.884, −0.837)	1.861	**0.042**	0.289
Vitamin B9	−1.728 (−4.751, 0.216)	1.346	0.207	0.193	−2.819 (−6.466, 0.090)	1.746	0.137	0.247
Calcium	−2.855 (−5.739, 0.539)	1.525	0.077	0.238	−5.603 (−11.506, 0.341)	2.679	0.074	0.342
MUFA	0.531 (−1.632, 2.577)	1.139	0.648	0.159	−2.158 (−6.467, 1.069)	1.721	0.255	0.227
Physical activity	−0.032 (−2.535, 2.394)	1.266	0.977	0.152	−3.731 (−7.486, −0.200)	1.586	0.059	0.270
Pro-oxidants								
Fer	2.439 (0.066, 4.682)	1.318	0.083	0.221	3.637 (0.365, 7.643)	2.073	0.115	0.266
SFA	−2.489 (−5.088, 0.176)	0.185	0.056	0.233	−1.357 (−5.860, 2.519)	2.043	0.513	0.213
PUFA	−1.826 (−6.053, 2.704)	2.222	0.412	0.170	2.335 (−2.417, 8.302)	2.043	0.513	0.213
Alcohol	−1.114 (−3.616, 1.298)	1.232	0.381	0.174	−0.917 (−5.101, 2.424)	2.010	0.662	0.209

The regression coefficients (β), the corresponding 95% confidence intervals (CI) and standard errors (SEs), and adjusted R^2^ are shown (representing the proportion of variance explained). Bold indicates statistical significance at *p* < 0.05. Abbreviations: ALT, alanine aminotransferase; AST, aspartate aminotransferase; SFA, Saturated fatty acid; PUFA, Polyunsaturated fatty acids. Model is adjusted for age, sex, and clinical subgroup.

## Data Availability

All data related to this work are presented in the article.

## Supplementary Materials

The following supporting information can be downloaded at: https://www.mdpi.com/article/10.3390/antiox15091077/s1, Table S1: Oxidative balance score (OBS) point assignment scheme; Table S2: Quantitative summaries of multicollinearity and influential observations for multiple linear regression model 1 and 2; Table S3: Pharmacological treatment and medication supplement of study participants.
