# Supplementary material for: Association of Nutrient-, Food-, and Lifestyle-Based Oxidative Balance Scores with Liver Enzymes in Older Adults Across Cardiovascular-Risk Groups
Source: Antioxidants (Basel). 2026 Aug 28;15(9):1077. doi: 10.3390/antiox15091077 (PMC13603438; doi:10.3390/antiox15091077)
Supplement: Supplementary file 1 [file antioxidants-15-01077-s001.zip › antioxidants-4454501-supplementary.pdf]

antioxidants

MDPI

Supplementary Table S1. Oxidative balance score (OBS) point assignment scheme

OBS Components	Score assignment scheme
**Antioxidants**	
**Nutrient**	
Vitamin C (%RDA)	0 = low (1st tertile), 1 = medium (2nd tertile); 2 = high (3rd tertile)
Vitamin E (%RDA)	0 = low (1st tertile), 1 = medium (2nd tertile); 2 = high (3rd tertile)
Vitamin A (%RDA)	0 = low (1st tertile), 1 = medium (2nd tertile); 2 = high (3rd tertile)
MUFA (% energy)	0 = low (1st tertile), 1 = medium (2nd tertile); 2 = high (3rd tertile)
Fibres (%AI)	0 = low (1st tertile), 1 = medium (2nd tertile); 2 = high (3rd tertile)
Selenium (%RDA)	0 = low (1st tertile), 1 = medium (2nd tertile); 2 = high (3rd tertile)
Zinc (%RDA)	0 = low (1st tertile), 1 = medium (2nd tertile); 2 = high (3rd tertile)
Vitamin B9 (%RDA)	0 = low (1st tertile), 1 = medium (2nd tertile); 2 = high (3rd tertile)
Calcium (%RDA)	0 = low (1st tertile), 1 = medium (2nd tertile); 2 = high (3rd tertile)
ß-carotene (µg /d)	0 = low (1st tertile), 1 = medium (2nd tertile); 2 = high (3rd tertile)
Lutein/ zeaxanthin (µg/d)	0 = low (1st tertile), 1 = medium (2nd tertile); 2 = high (3rd tertile)
Lycopene (µg/d)	0 = low (1st tertile), 1 = medium (2nd tertile); 2 = high (3rd tertile)
**Food**	
Vegetables (g/d)	0 = low (1st tertile), 1 = medium (2nd tertile); 2 = high (3rd tertile)
Fruits (g/d)	0 = low (1st tertile), 1 = medium (2nd tertile); 2 = high (3rd tertile)
Legumes (g/d)	0 = low (1st tertile), 1 = medium (2nd tertile); 2 = high (3rd tertile)
Fish (g/d)	0 = low (1st tertile), 1 = medium (2nd tertile); 2 = high (3rd tertile)
Nuts (g/d)	0 = low (1st tertile), 1 = medium (2nd tertile); 2 = high (3rd tertile)
**Lifestyle**	
Physical activity	Male: 0 = 1.3 PAL, 1 = 1.11 PAL, 2 = + 1.25 PAL<br>Female: 0 = 1.3 PAL, 1 = 1.12 PAL, 2 = + 1.27 PAL
**Pro-oxidants**	
**Nutrient**	
Iron (%RDA)	0 = high (3rd tertile), 1 = medium (2nd tertile), 2 = low (1st tertile)
PUFA (% energy)	0 = high (3rd tertile), 1 = medium (2nd tertile), 2 = low (1st tertile)
SFA (% energy)	0 = high (3rd tertile), 1 = medium (2nd tertile), 2 = low (1st tertile)
**Food**	
Red and processed meat (g/d)	0 = high (3rd tertile), 1 = medium (2nd tertile), 2 = low (1st tertile)
Refined grains (g/d)	0 = high (3rd tertile), 1 = medium (2nd tertile), 2 = low (1st tertile)
Sweets (g/d)	0 = high (3rd tertile), 1 = medium (2nd tertile), 2 = low (1st tertile)
Salty snacks (g/d)	0 = high (3rd tertile), 1 = medium (2nd tertile), 2 = low (1st tertile)
**Lifestyle**	
Alcohol (g/d)	0 = high (3rd tertile), 1 = medium (2nd tertile), 2 = low (1st tertile)

Abbreviations: SFA, Saturated fatty acid; MFA, Monounsaturated fatty acids; PUFA, Polyunsaturated fatty acids; PAL, Physical activity level

Supplementary Table S2. Quantitative summaries of multicollinearity and influential observations for multiple linear regression model 1 and 2

	OBSN			OBSN-L		
	Tolerance range	VIF range	Cook's distance range	Tolerance range	VIF range	Cook's distance range
ALT	0.808-0.999	1.001-1.047	0.000-0.521	0.852-0.999	1.001-1.044	0.000-0.563
AST	0.808-0.999	1.001-1.047	0.000-0.207	0.852-0.999	1.001-1.044	0.000-0.222

Tolerance and VIF values were used to assess multicollinearity, whereas Cook's distance was used to identify potentially influential observations. No evidence of problematic multicollinearity or highly influential observations was detected (all VIF < 1.05 and Cook's distance < 1).

Abbreviations: ALT, alanine aminotransferase; AST, aspartate aminotransferase.; VIF, variance inflation factor.

Supplementary Table S3. Pharmacological treatment and medication supplement of study participants.

Discharge Medications, n (%)	*Healthy* (n =16)	Hypercholesterolemi (n = 14)	Post-MI (n=14)
**Antiplatelet Agents**			
Aas ec (Aspirin)+Brilinta<br>Clopidogrel<br>Asaphen ec	0	0	14 (100%)
**Anti-inflammatory agent:**			
Jamp colchicine<br>Pulmicort + Symbicort	0	0	5 (35.7%)
**Beta-Blockers**			
Bisoprolol<br>Carvedilol<br>Metoprolol	0	0	9 (64.3%)
**ACE Inhibitors (ACEi)**			
Lisinopril<br>Trandolapril<br>Perindopril<br>Ramipril	0	0	11 (78.6%)
**Hypoglycaemic**			
Forxiga	0	0	1 (7.1%)
**Anticoagulant**			
Eliquis	0	0	2 (14.3%)
**Statin therapy**			
Atorvastatin	0	0	11 (78.6%)
Rosuvastatin	0	0	3 (21.4%)
**Others**			
Vitamins (D, B-12)	0	0	5 (35.7%)
Calcium supplement	0	0	2 (14%)
